# Address before the Graduating Class of the Buffalo Medical College, February 24th, 1874

**Published:** 1874-03

**Authors:** Joseph Warren


### Miscellaneous.

### ADDRESS.

Delivered by JOSEPH WARREN, Esq., before the Graduating Class of the Medical Department of the University of Buffalo, at the Twenty-eighth Annual Commencement, Feb. 24, 1874.

The invitation which brings me here this evening, though it originated in pleasantry, was not extended because I am a doctor, or the son of a doctor, but because from long familiarity with physicians—in altogether a social way—I have acquired the dangerous habit of talking. The more persistent cultivation of the talent for science would have been better for you and better for me.

There is no reason, however, why any man trained to thought and action should not address his fellows upon any subject which relates to the progress of science and the welfare of the community. Coleridge divides the series of admirable essays entitled "The Friend" into three parts, which he designates as "Landing Places," and in explanation of this term says: "Among my earliest impressions I still distinctly remember that of my entrance into the mansion of a neighboring baronet, awfully known to me by the name of the great honse, its exterior having been long connected in my childish imagination with the feelings and fancies stirred up in me by the perusal of the Arabian Nights Entertainments. Beyond all other objects, I was most struck with the magnificent staircase, relieved at well-proportioned intervals by spacious landing-places—this adorned with grand or showy paints; the next looking out on an extensive prospect through the stately window, with its side-panes of rich blues and saturated amber or orange tints; while from the last and highest the eye commanded the whole spiral ascent with the marble pavement of the great hall from which it seemed to spring up as if it merely used the ground on which it rested."

Young gentlemen of the Graduating Class, you are standing to-night upon the first landing-place of the grand staircase of professional and public life. Whether you are to go up higher, to be men of broader grasp and finer culture, to become masters of science and lovers and practitioners of art, is to be determined by yourselves. The Buffalo Medical College has presented you with the seal of its commendation and its sanction for entrance upon a career of usefulness. The measure of the debt of gratitude you owe to your instructors is known to you, and this can only be paid by contributing in turn your share to the building up of the College. The pride of the mother is the character of her son, and it is the sacred duty of the son to reflect glory on the mother. The Buffalo Medical College is your professional *Alma Mater*—

cherishing mother—who sends you from home with her smile and benediction, confidently expecting that you will be worthy of her and her profession.

While on this landing-place, you will pardon me for presenting a brief history of this College so that you may learn whether or no you should be proud of the honor of the degree conferred upon you.

The charter for the University of Buffalo, of which this College is yet the only organized department, was obtained from the legislature in the winter of 1845–46, mainly through the exertions of the Hon. N. K. Hall, who was then a member of the Assen bly from this city; the most active promotors of the enterprise being Drs. Austin Flint and James P. White. As has since occasionally happened in Buffalo, more or less oppositiou was manifested to this new departure. It was seriously intimated that the young physicians and their confederates were not altogether unselfish in their desire to establish a College of Medicine; that they contemplated some financial gain or the improper increase of their professional practice. If the unwritten history of that time has not been incorrectly handed down, there was more or less bandying of unprofessional epithets and fierce display of professional implements of warfare The College was not abandoned on this account. The first lectures—in the spring of 1847, in the winter of 1847 and 1848, and of 1848 and 1849—were given in the wooden building on the corner of Washington and Seneca streets, over the old Post-office and where the present Post-office now stands.

The following gentlemen composed the first faculty :

*Chemistry and Pharmacy*—Jas. Hadley, M. D.

*Physiology and Medical Jurisprudence*—Charles B. Coventry, M. D.

*General and Special Anatomy*—James Webster, M. D.

*Pathology and Materia Medica*—Charles A. Lee, M. D.

*Principles and Practice of Surgery aud Clinical Surgery*—Frank H. Hamilton, M. D.

*Obstetrics and Diseases of Women and Children*—James P. White, M. D.

*Principles and Practice of Medicine and Clinical Medicine*—Austin Flint, M. D.

It should however, be stated that Prof. Geo. Hadley son of Prof. Jas. Hadley, has filled acceptably the chair of chemistry from the first.

The Buffalo *Medical Journal* of September, 1846, in calling attention to the contemplated college, states that the city has nearly thirty thousand population, and that it possesses advantages for the practical study of medical science that cannot be claimed for smaller places.

The First Commencement exercises were held on the 16th of June, 1847, at the First Presbyterian church, the Rev. Dr. Hopkins making the opening prayer. The degree of Doctor of Medicine was conferred upon seventeen young gentlemen by the Hon. Millard Fillmore, Chancellor of the University, and addresses were made by Mr. Fillmore and Prof. Hamilton.

The Second Commencement took place in May, 1848, at the Washington street Baptist church. Thomas M. Foote, M. D., in the absence of Mr. Fillmore, conferred the degrees upon thirty-two graduates, and Dr. Austin Flint delivered the address.

The Third Commencement took place on the 18th of April, 1849, at the First Presbyterian church. The address was delivered by the Rev. M. L. R. P. Thompson to the nineteen graduates. In this year the construction of the present Medical College building was commenced. The enterprise was already a demonstrated success, and the public spirited citizens of that day determined to give it a local habitation. The subscription list is an interesting fragment of local history. The largest donors were the late Jesse Ketchum, and A. D. Patchin, who gave $600 and $500 respectively ; the late Hon. A. H. Tracy, George W. Tifft and E. G. Spaulding, who gave $200 each, and then follow the names of eighty or more gentlemen who gave $100 or less. In such sums as these $8,000 was obtained ; the State gave $2,000 and $5,000 remained on bond and mortgage. The cost of the lot and building was about $15,000—an amount too insignificant to engage the attention of a city contractor now-a-days, and yet it was sufficient to construct in the most thorough and substantial manner a commodious and well appointed edifice which has preserved the freshness of youth during all these years. This College building was opened in 1849, with a suitable congratulatory address by Dr. Austin Flint.

The Fourth Commencement occurred March 27th, 1850. Hon. Geo. W. Clinton conferred the degrees on twenty-seven graduates, and Dr. James P. White delivered the address.

The Fifth Commencement was on the 26th of February, 1851, Dr. Thomas M. Foote conferring the degrees upon thirty graduates, and Prof. Charles B. Coventry delivering the address. Among the graduates of this year were the late Sandford Eastman and Albert J. Myer, Chief Signal Officer of the United States Army, who has identified himself as thoroughly with the history of the country as any man living or dead. His system of signals utilized in the war of the rebellion and the system of the weather observations and reports already established and destined to be still farther developed and adopted over the civilized world, give him and the College unchallenged distinction. Of Dr. Eastman I shall have opportunity to speak hereafter.

Before the next course of lectures, the first change in the faculty of the College is recorded. Prof. Palmer accepted the Chair of Anatomy made vacant by the resignation of Prof. Webster, who was, as I learn, a skillful master of the scalpel and a very fluent and successful lecturer. In the course of this year, too, John C. Dalton, now Professor of Physiology in the College of Physicians and Surgeons of the City of New York, commenced a series of lectures on Physiology, in which vivisection was practically introduced for the first time in the United States. He repeated and improved upon the experiments upon living animals made by Sir Charles Bell, Magendie and Bernard, and demonstrated the functions of the vital organs of the human system. The result of this method of demonstrative teaching is recognized in the more thorough knowledge of the offices of the heart, liver and the pancreas, and of the diseases which are referable to the spinal cord and the nervous and respiratory system. Prof Dalton has since published a book on Physiology, which has become the text book of all the schools.

The Sixth Commencement was celebrated on the 25th of April, 1852, Hon. James Wadsworth then, Mayor, conferring the degrees upon twenty graduates to whom Prof. Chas. A. Lee made the address.

The Seventh Commencement was on the 27th of April, 1853, Hon. George W. Clinton conferring the degrees upon eighteen graduates, and Dr. Hamilton delivered the address, During this year Dr. Sanford B. Hunt became connected with the college as Demonstrator of Anatomy.

Before the beginning of the next course of lectures Dr. Thos. F. Rochester was called to the Chair of the Theory and Practice of Medicine, made vacant by the resignation of Dr. Flint, who accepted another field of professional labor. As has been before stated, Dr. Flint was one of the promoters of the college, and his instruction did much to establish and maintain its highest reputation. Since this time as a physician and an author of works on General Practice and Diseases of the Heart and Lungs he has achieved a world-wide reputation and stands confessedly at the head of his profession in the United States.

The Eighth Commencement took place Feb. 22, 1854, Chancellor Fillmore conferring the degrees upon twenty-four gentlemen, and Dr. Rochester delivering the address. This year Dr. S. B. Hunt was appointed to the chair of Anatomy, made vacant by the resignation of Prof. Palmer, and Dr. Boardman succeeded as Demonstrator of Anatomy.

The Ninth Commencement was held at American Hall, Feb. 22, 1855, Chancellor Fillmore conferring the degrees upon fourteen gentlemen, and Dr. Hunt delivering the address.

The Tenth Commencement took place at Kremlin Hall. Feb. 26, 1856, Judge Clinton conferring the degree upon graduates, and Professor James P. White pronouncing the address.

At the Eleventh Commencement, Feb. 25th, 1857, Chancellor Fillmore conferred the degrees upon fifteen graduates, and Prof. Austin Flint, who had returned and resumed his connection with the College as Professor of Clinical Medicine, delivered the address.

At the Twelfth Commencement, Feb. 22d, 1858, the Chancellor conferred the degrees upon nine graduates, who were addressed by Prof. Moore, of Rochester.

Before the next course of lectures the Faculty made arrangements to introduce new features in the instruction. Dr. Theophilus Mack, of St. Catherines, was appointed lecturer on Materia Medica and Therapeutics; Dr. Sandford Eastman was appointed Professor of Anatomy in place of Dr. Hunt, who had renounced the teaching and practice of medicine for the more congenial labors of the editorial profession; and Dr. Austin Flint, Jr., was appointed Professor of Physiology and Microscopic Anatomy. In this connection, it is due to Dr. Hunt to say that both by his editorial management of the Buffalo *Medical Journal* and his admirable lectures in his department, he contributed largely to the prosperity of the College.

Dr. Flint took up with enthusiasm and prosecuted with marked success the teaching of Physiology by vivisection, introduced by his preceptor, Dr. Dalton. His experiments showing the method of the transfusion of blood, are spoken of as remarkably brilliant, and his lectures were more than usually interesting and instructive. Since this, he has become the teacher of Physiology in the Bellevue Medical College, and has written the most comprehensive and exhaustive work on physiological science in the English language.

The Thirteenth Commencement took place Feb. 22, 1859, Chancellor Fillmore conferring the degrees upon thirteen graduates, and Prof. Hamilton delivering the address.

At the Fourteenth Commencement, Feb. 21, 1860, the Chancellor conferred the degrees upon twenty-three graduates, and Dr. Theophilus Mack, of St. Catherines, delivered the address.

At the Fifteenth Commencement, Feb. 26, 1861, the Chancellor conferred the degrees upon thirty graduates, and the address was delivered by Prof. Joshua R. Lothrop, who had taken the place of Dr. Mack.

At the Sixteenth Commencement, Feb. 25, 1862, twenty-eight students were graduated, Prof. James P. White delivering the address.

During this year, Dr. W. H. Mason succeeded Dr. Austin Flint, Jr., as Professor of Physiology, and still retains the position, carrying forward the practice of experimental teaching of his predecessor, and adding something every year to the treasures of knowledge which make medicine every year more of a science. Not long since an example of the experiments made by Dr. Mason was related to me, for the correctness of which I do not vouch. All the blood was taken from a dog and carefully preserved at the proper temperature. Artificial respiration was maintained until the veins of another dog could be opened, when the blood of dog

No. 2 was pumped into dog No. 1, much to the delight of the almost defunct canine who revived and went about his business. The blood of dog No. 1 was then transmitted to dog No. 2, and he, too, was alive again and well. Whether both these animals are now in the enjoyment of perfect health or not I am unable to say; but it would not surprise me to learn that the small dog had died of apoplexy and the larger one of exhaustion.

At the Seventeenth Commencement, held February 24, 1863, twenty-four students were graduated, Dr. Rochester delivering the address; at the Eighteenth, February 23, 1864, forty-one students took their degrees, Prof. Charles A. Lee delivering the address; at the Nineteenth, Feb. 21, 1865, fifty students were proclaimed doctors, Prof. Moore making the address; at the Twentieth, Feb. 20, 1866, the graduates numbered forty, and Right-Rev. Bishop Coxe was the orator of the evening; at the Twenty-first, Feb. 26, 1867, the graduates were forty, and Dr. James P. White delivered an address, largely historical, in which he said: "In many things essential to an elevated standard of medical education the founders of this College claim to have led the way in reform and improvement. It has ever been, also, the desire of those to whom the interests of this institution have been confided, to send forth such alumni only as would do honor to that profession to which its credentials might form a passport. As an evidence of the thoroughness of the course of instruction in this institution, the faculty are gratified in being able to assert that among all the candidates who presented themselves during the war for examination before the medical boards of the different States, or the more exacting United Army Board, or the still more severe scrutiny of the medical staff for the Navy, they have yet to learn of the first rejection of one holding its degree of Doctor in Medicine."

The twenty-second Commencement occurred February 25, 1868. The graduates were forty, and the address was delivered by Dr. J. F. Miner, who had this year been appointed Professor of Surgical Anatomy and Ophthalmology, changed in 1869 to Special Surgery.

At the Twenty-third Commencement, Feb. 24, 1869, thirty-four students were graduated—address by Prof. Rochester; at the Twenty-fourth, Feb, 22, 1870, the graduates were forty-one, and the orator Rev. Walter Clark, D. D.; at the Twenty-fifth, Feb. 20, 1871, the graduates were thirty-nine, Prof. Moore, of Rochester, delivering the address. In consequence of the illness of Dr. Eastman, Dr. M. G. Potter delivered during this term the lectures on Anatomy. At the Twenty-sixth, Feb. 20, 1872, the graduates were thirty-four, and Prof. White delivered the address. During the year Prof. Chas. A. Lee, who had been identified with the College from the outset, died. As a lecturer in several medical colleges; as editor of the New York *Journal of Medicine;* as the author of popular works on hygiene and sanitary subjects, and as the editor of a new edition of Copland's Medical Dictionary with copious explanatory notes and additions, Prof. Lee was widely and favorably known. He was a man of culture, a lover of science for its own sake, and a valuable contributor to all the departments of medical learning. Dr. H. N. Eastman, of Geneva, lectured in this department during this term and the next. Dr. Potter was elected Prof., of Anatomy during this year in place of Dr. Eastman.

The Twenty-seventh Commencement exercises were held on the 25th of February, 1873. Professor J. F. Miner delivered the address to the forty graduates. During this year, Dr. Stoddard, of Rochester, was appointed Lecturer on Materia Medica, and Dr. Phelps, Demonstrator of Anatomy.

If this recital of facts and dates has not been too wearisome, you will have already arrived at the conclusion that an institution with such a history—with such a distinguished list of eminent teachers —with so many and accomplished graduates, is one whose honors should be worthily borne. Among the surgeons of the country, Dr. F. H. Hamilton, now of New York, Dr. Edwin M. Moore, of Rochester, and Dr. Miner, of this city, have no superiors; among the general practitioners, Drs. Austin Flint, Sr., and Rochester hold conceded eminence; among the physiologists, Drs. Dalton, Austin Flint, Jr., and Mason are in the first rank, and Dr. White in his department has initiated and performed operations which have won for him a reputation more than national, and contributed largely to the progress of medical science.

The second landing-place, Gentlemen, of the Graduating Class, you will remember looked "out on an extensive prospect through the stately window, with its side panes of rich blues and saturated amber or orange tints." To this landing.place you will ascend, if at all, by slow and patient steps. The stair-case is before you, the stately window with its beautiful tints is above. If you anticipate that some stroke of fortune is to give you precedence; that some accident is to elevate you above your associates, it is more than probable that you will taste the sorrows of disappointment. To a greater or less extent you are scientific men, and it now devolves upon you to demonstrate that you have the wisdom to make your knowledge of the science of medicine available. It is not enough that you are capable of healing the sick. You must be able to persuade the citizens of your residence of your capacity and skill. Many a well educated physician, enthusiastic in the love of his profession, has failed because to his other learning he failed to add that of a correct diagnosis of human nature. You cannot be successful as practitioners if you isolate yourself from society and ignore the culture which grows out of contact with well-informed and well-balanced minds. So far as my observation has extended, physicians may be divided into three classes:

1. Those who gain strength, steadiness and practical knowledge every year, and add to their professional reputation the element of high moral character.

2. Those who by some spasmodic effort—some assumption of superior wisdom or special skill—attempt to reach the second landing-place without the use of the stairs leading to it.

3. Those who wait for something to turn up, and gradually slide or fall from the first landing-place in the bottom of the staircase.

The first class comprises the growing and great men of the medical profession; the second the empirics and quacks who are as emphemeral as they are useless, and the third are the drones and dullards that afflict every community. Permit me to call your attention to a few maxims which may be of value in professional life:

1. Be cleanly in person and neat in dress. Patients never call a doctor because he needs their patronage.

2. Sometimes tell the truth to your patients. It will surprise and may benefit them.

3. Associate with the most reputable people of your locality, or cultivate your own acquaintance,

4 If you hear any scandal, keep it to yourself; if you learn anything good of another, tell it.

5. Avoid people who are constitutional fault-finders, and strive to gain the confidence of those who are in favor of something.

6. If you can, secure business on the Chinese system of payment when the family is in perfect health, and "no pay" when any member is ill. It will be easier and safer to prevent disease than to cure it.

7. Bear in mind that the symptomes of a cold are, also, the symptoms of nearly all the types of febrile and inflammatory disease. The best thing for the patient and the worst for the physician, is to break up the cold and drive off the contingent fever. This is announced on the authority of the late Dr. Barnes of this city.

8. A most desirable attainment for a physician is composure. Said David Paul Brown to a gentleman who inquired how his son could become self-poised: "That must depend upon himself. He must learn composure as Peter the Great learned to conquer, by being flogged and defeated over and over again, deriving instruction from every overthrow In short, he must let no man be master of his temper but himself."

These maxims could easily be extended, but remembering that

——Since brevity is the soul of wit,
And tediousness the limbs and outward flourishes,

I will be brief.

In conclusion, Gentlemen of the Graduating Class, I ask your permission to pay a personal tribute to the memory of my more than friend, the late Dr. Sandford Eastman. He was a graduate of this college and was called to a prominent professorship. Thoroughly scientifice and eminently practical, he discharged his duties to the college as he did those of every department of life with singular fidelity. He lectured conscientiously, not to gain popular applause, nor even class favor. His aim was simply to do, day by day, his best for his profession, and for all those who came within the range of his personal and professional knowledge. He watched by the sick-bed of the poor without hope of reward; he prayed with and for those near unto death to whom the church brought no benediction; he lingered with the sorrowing and suffering when life had fled, and ministered to them as only the sympathetic spirit can; like his Master, he went about doing good, and the consciousness of well-doing was to him its own sufficient reward. Human and manly as he was, he may have had dreams of higher recognition in a professional way; but they did not disturb the even tenor of his patient though waning life. Jacob's ladder was always near him, it was but a step or two between each day's work and Heaven; and angels were constantly ascending and descending.

Some two years ago, as some of you know, he resigned his professorship and sought a more genial climate. The skies of Southern California smiled him welcome—the dryer air gave him vigor and encouragement—the blossoms of the orange and of all the plants of perfume were brighter and sweeter for his coming. He thought to live awhile, until his daughters were educated and the orange grove he had planted should bear golden fruit; and yet the call of the sick could not be resisted. He must be about the Master's business, and so, on the 8th of January last, having finished his toil upon earth, and gladly aspiring to the Landing Place of Heaven, he ascended the steps and rests from his labors. Looking down from his celestial home, he surely asks these graduates of the Buffalo Medical College, as those who have preceded them, to deserve the title of "The Beloved Physician."

